# Indirect Protection from Vaccinating Children against Influenza A Virus Infection in Households

**DOI:** 10.3390/v14102097

**Published:** 2022-09-21

**Authors:** Tim K. Tsang, Can Wang, Vicky J. Fang, Ranawaka A. P. M. Perera, Hau Chi So, Dennis K. M. Ip, J. S. Malik Peiris, Gabriel M. Leung, Simon Cauchemez, Benjamin J. Cowling

**Affiliations:** 1 WHO Collaborating Centre for Infectious Disease Epidemiology and Control, School of Public Health, Li Ka Shing Faculty of Medicine, The University of Hong Kong, Hong Kong, China; 2 Laboratory of Data Discovery for Health Limited, Hong Kong Science and Technology Park, New Territories, Hong Kong, China; 3 HKU-Pasteur Research Pole, The University of Hong Kong, Hong Kong, China; 4 Mathematical Modelling of Infectious Diseases Unit, Institut Pasteur, UMR2000, CNRS, 75015 Paris, France

**Keywords:** influenza, vaccination, indirect protection

## Abstract

Influenza vaccination is an important intervention to prevent influenza virus infection. Our previous analysis suggested that indirect protection is limited in an influenza B epidemic in Hong Kong. We further analyzed six influenza A epidemics to determine such potential. We applied a statistical model to estimate household transmission dynamics in the 3 influenza A(H3N2) and 3 pandemic influenza A(H1N1) epidemics. Then, we estimated the reduction in infection risk among unvaccinated household members when all children in households are vaccinated, with different assumptions on vaccine efficacy (VE). In the optimal scenario that VE was 70%, the reduction to the total probability of infection was only marginal, with relative probabilities ranged from 0.91–0.94 when all children in households were vaccinated because community was by far the main source of infection during the six epidemics in our study. The proportion of cases attributed to household transmission was 10% (95% CrI: 7%, 13%). Individual influenza vaccination is important even when other household members are vaccinated, given the degree of indirect protection is small.

## 1. Introduction

Influenza causes substantial morbidity and mortality in humans each year on average [1,2]. Previous analyses suggest that children have the highest risk of infection [3,4], while the elderly have the highest risk of more severe disease after infections [5,6]. Transmission of influenza virus is thought to occur in different settings, including households, schools, and workplaces [7,8,9].

Vaccines have been available since 1945 to prevent infection, since then it is an important tool to prevent infection and transmission of influenza viruses. Direct protection of vaccination (the reduction of infection risk for vaccinee after vaccination) is clear [10,11], but the degree and public health significance of indirect protection of vaccination (the reduction of infection risk in unvaccinated people if their contacts are vaccinated) are less clear [12]. Herd immunity could be achieved by having large scale vaccination campaigns targeting a large fraction of high-risk groups such as children [13,14,15]. However, at a smaller scale, such impact is less clear. In our previous analyses, we estimated that the indirect protection in influenza B epidemics is limited, in the sense that the infection risk of unvaccinated household members may be reduced by <10% when one child was vaccinated [16]. Furthermore, two small household studies suggested the potential of indirect protection [17,18], but another one reported no such protection [19]. Given that children are believed to be the important drivers of influenza transmission, it is important to clarify the potential of such protection [20,21].

Here, we use a previously developed directed graph model to estimate the dynamics of influenza A virus transmission in households, to analyze data from a household cohort study from 2009–2013, in which covered 6 influenza A epidemics [22]. Then, we use the estimated transmission dynamics to quantify the degree of indirect protection from vaccinating children in households.

## 2. Methods

### 2.1. Study Design

Data were collected in two community-based randomized controlled trial (ClinicalTrials.gov NCT00792051) aiming to evaluate the direct and indirect benefits of influenza vaccination [22,23]. In the two studies, we recruited 119 and 796 households with at least one child in 2008/09 and 2009/10. One child aged 6–17 in each household was randomly selected to receive trivalent inactivated influenza vaccination or saline placebo. Participants provided serum specimens at the start of the study, and after 6 and 12 months. After the first year, all households were invited to participate in the follow-up observational study from 2009 to 2013 without intervention [24]. In each year, participants provided serum specimens in autumn (Oct to Dec), and also spring (April–May). We also obtained annual records of influenza vaccination from all participants.

### 2.2. Laboratory Methods

All serum specimens were tested in parallel for antibody responses by hemagglutination inhibition (HAI) assays in serial doubling dilutions, starting from an initial dilution of 1:10 using standard methods [25]. Antibody titer was defined as the reciprocal of the highest dilution of serum that prevents complete hemagglutination wells. In the pilot (2008/09), serum specimens were tested against A/California/7/2009(H1N1) and A/Brisbane/10/2007(H3N2). In the main trial and second year of follow-up, i.e., 2009/10 and 2010/11, serum specimens were tested against A/California/7/2009(H1N1) and A/Perth/16/2009-like (H3N2). In 2011/12 and 2012/13 serum specimens were tested against A/California/7/2009(H1N1) and A/Victoria/361/2011-like (H3N2).

### 2.3. Model Details and Inference

We followed our previously developed statistical framework to estimate the probability of infection acquired from the community during the epidemic period and the probability of household transmission from the serologic data, to overcome the challenge that the chains of transmission are unobserved and only the final infection status of each individual at the end of the epidemic is available [20,26,27,28]. The method was based on directed graphs (digraph) described in detail in Cauchemez et al. [20] and summarized in Supplementary Methods. In short, each household member was represented by a vertex, and hence a random directed graph with *n* vertices represented a household of size *n*. Possible transmission events were represented by edges. If individual *i* would get infected when individual *j* was infected, then an edge was added between individual *j* and individual *i*. An edge between the community and individual *i* indicated that individual *i* was infected. Hence, denote variable $v^{ji}$ the presence of an edge from individual *j* to individual *i*, occurring with the following probability:

$$P\left(v^{ji}=1|\theta \right)=1-\exp\left(-\lambda^{ji}\left(\theta \right)\right)$$

The formulation of $\lambda^{ji}\left(\theta \right)$ is as follows:

$$\lambda^{ji}\left(\theta \right)=\left\{\lambda_{h1}^{}I\left(hs<4\right)+\lambda_{h2}^{}I\left(hs\geq 4\right)\right\}*S_{k}\left(\theta \right),$$

where$\;\lambda_{h1}^{}$,$\;\lambda_{h2}^{}$ are model parameters for the transmission in households of size <4 and ≥4, respectively, and $S_{k}\left(\theta \right)$ is the susceptibility variable for individual *k* described in Supplementary Materials; Section 1.1.5.

We conducted our analysis in a Bayesian framework, and we considered the digraph as augmented data given that the transmission chains were unobserved. We applied a data augmentation Markov chain Monte Carlo approach to jointly explore the parameters and digraph space, and estimate the posterior distribution of the model parameters [20,26] (Supplementary Materials).

### 2.4. Model Specification

In our study, infection was defined as 4-fold or greater rise in paired sera. Vaccinated individuals were excluded (assumed infection status to be missing), since vaccination could cause 4-fold or greater rise in HAI titer, which was not distinguishable from infection. Therefore, we first fitted the model to the data to describe the household transmission dynamics. Then, we used the fitted model to predict the degree of indirect protection.

Hence, other possible factors that may affect the transmission dynamics in households were added, including, age groups (0–11, 12–17, 18–44, 45–64 and 65+) and level of pre-season HAI titers. Children and adults were defined as individuals ≤18 and >18 years of age. A higher pre-season HAI titer may provide protection against infection [29]. We also tested models with difference in infectivity between children and adults, and models with different transmission probability in households with household size 2–3 or 4+.

To estimate the proportion of cases attributed to household transmission, a previous approach described by Cauchemez et al. was used [20]. For a parameter vector from the posterior distribution, we could simulate epidemics in households with the household transmission parameters unchanged or set to zero. This proportion was the difference in case counts between these two scenarios.

### 2.5. Model Adequacy and Comparison

Model adequacy was assessed by comparing the observed and expected number of infections in households (Supplementary Materials). We used Deviance Information Criterion (DIC) to compare models [30]. Smaller DIC indicated a better model fit, and DIC difference >5 was considered as a substantial improvement [31]. DIC cannot be directly computed for a given model [32] due to the unavailability of the likelihood of observed data. Hence, an importance sampling approach was used to estimate the likelihood for the observed data and evaluate the DIC [20,33] (Supplementary Materials). 

### 2.6. Model Prediction

A simulation study was conducted to evaluate the indirect protection from influenza vaccination. We explored two vaccine strategies, 1: vaccinating one child in each household, 2: vaccinating all children in each household. In this simulation study, 10,000 epidemics were simulated. In each simulation, there were 150,000 households, and the model parameters drawn from their posterior distributions. 

In the simulation, the corresponding digraphs were recorded. Therefore, the source of each infection could be determined, and the probability of infection from the community and households could be estimated. We used the previous approach [16] to impute the inconclusive source by assuming half of them were infected from the community. The indirect protection due to a vaccine strategy can be quantified by the ratio of the probability of infection in a group and from a source under a vaccine strategy, compared to the corresponding probability of infection under no vaccination strategy (Supplementary Materials). 

### 2.7. Sensitivity Analysis

In our main model, we assumed that the probabilities of household transmission were the same over seasons with the same subtype. While this was supported by household case-ascertain studies that these probabilities ranged from 5–15%, we conducted a sensitivity analysis that this assumption was relaxed (Supplementary Materials). 

## 3. Results

### 3.1. Study Participants

**Figure 1:**
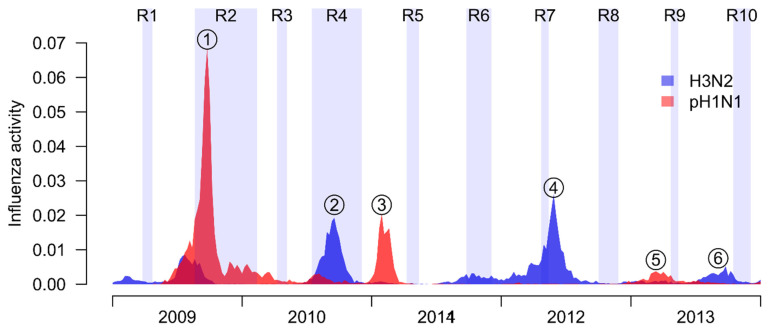
Timelines of our study. In our study, there were 10 rounds of sera collections (R1 to 10), which were represented by shaded regions. Local influenza activity in our study period, including pandemic A(H1N1) influenza virus (red), and seasonal A(H3N2) virus (blue). Circle with numbers indicate the epidemics analyzed in our study.

There were 10 rounds of sera collection in the study in 2009–2013 (Figure 1). Based on local surveillance data, 6 major influenza A epidemics were identified during the study period, including the pH1N1 pandemic outbreak in 2009, and two pH1N1 epidemics in 2011 and 2013, and three H3N2 epidemics in 2010, 2012 and 2013, respectively (Figure 1). In total, 829 households were recruited in two trials, with 86 of them participants in both. After excluding households with no participants with available infection status, we analyzed data from 2512 participants for the pH1N1 pandemic outbreak, and 1443–1943 participants in the other 5 epidemics (Table S1). The distribution of age and distribution of HAI titer among 5 epidemics were shown in Table S1. The infection risk for these 5 epidemics ranged from 7% to 24%. For the first wave of pH1N1 pandemic, all individuals had HAI titer less than or equal to 10, and the infection risk was much higher (38%). 

### 3.2. Household Transmission Dynamics

**Figure 2:**
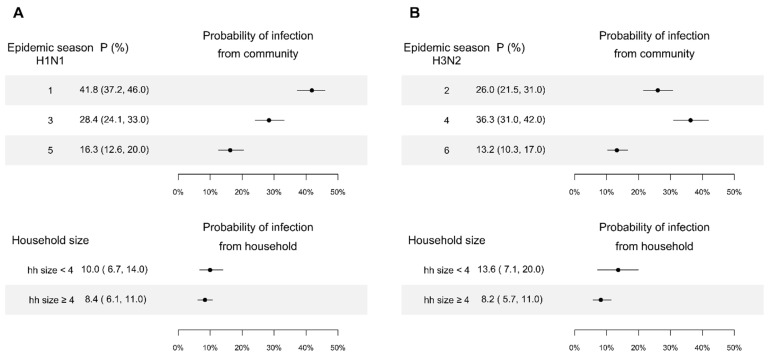
Probability of infection from community and person-to-person transmission in households for children aged less than 12, with a lower level of titer estimated by the digraph models. Point and line are the point estimate and their 95% credible interval. Panel (**A**): Estimates for pH1N1. Panel (**B**): Estimates for H3N2.

Based on model comparison, we found that models with age relative susceptibility, household size, and the protection of HAI titer is the best model (Table 1; Supplementary Figure S1). We estimated the probability of infection from the community for children aged less than 12 with a low level of HAI titer was 42% (95% Credible interval (CrI): 37%, 46%) in the first wave of pH1N1 pandemic, and 28% and 16% for subsequently two pH1N1 epidemics in 2011 and 2013 (Figure 2A). For H3N2, the probability of infection from the community for children aged less than 12 with a low level of HAI titer was 13–36% in three epidemics in 2010, 2012 and 2013 (Figure 2B). 

When exposed to an infected member in a household of size 2 or 3, children aged less than 12 with HAI titers <10 had a probability of infection of 10% (95% CI: 7%, 14%) and 14% (95% CI: 7%, 20%), for pH1N1 and H3N2, respectively, (Figure 2), while those in a household of size larger than or equal to 4 had a probability of infection of 8% (95% CI: 6%, 11%) and 8% (95% CI: 6%, 11%) respectively. Models ignoring the effect of household size performed substantially worse (∆DIC: 38.3).

**Figure 3:**
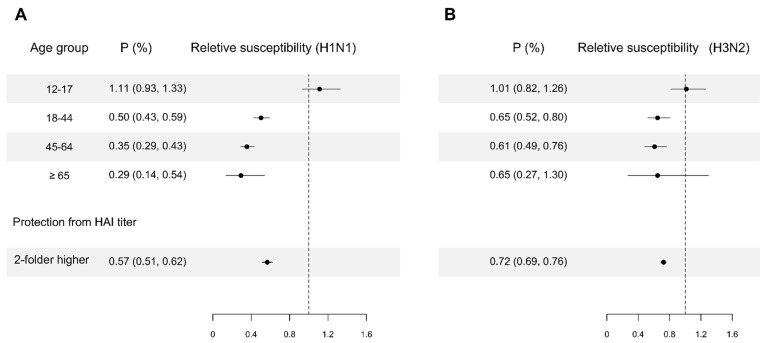
Estimates of relative susceptibility to infection for age groups and HAI titer. For age group relative susceptibility, the reference group of age is children less than or equal to 11 years of age. Panel (**A**): Estimates for pH1N1. Panel (**B**): Estimates for H3N2. Point and line indicate the point estimate and their 95% credible interval, which are constructed by using MCMC to fit the data with digraph model.

We estimated that adults and the elderly were less susceptible than children. For pH1N1, we estimated that adults aged 18–44, 45–64 and ≥65 were 50% (95% CrI: 41%, 57%), 65% (95% CrI: 57%, 71%) and 71% (95% CrI: 46%, 86%) less susceptible compared with children aged < 12. For H3N2, we estimated that adults aged 18–44 and 45–64 were 35% (95% CrI: 20%, 48%) and 39% (95% CrI: 24%, 51%) less susceptible compared with children aged < 12. Ignoring this difference in susceptibility by age groups substantially worsened model fit (∆DIC: 494.1). We estimated that every two-fold higher in HAI titer was associated with 43% (95% CrI: 38%, 49%) and 28% (95% CrI: 24%, 31%) lower susceptibility for pH1N1 and H3N2, respectively (Figure 3). The model without protection effect from pre-season HAI titers performed substantially worse (∆DIC: 372.9).

For the 6 influenza A epidemics in Hong Kong, we estimated that the proportion of cases attributed to household transmission was 10% (95% CrI: 7%, 13%). Models assuming difference in infectivity between children and adults performed substantially worse (∆DIC: 30.3).

### 3.3. Indirect Effect of Vaccination

Based on the fitted transmission model, we evaluated the effect of two vaccination strategies (strategy 1: vaccinate one child in each household and strategy 2: vaccinate all children in the household) on the probability of infection for unvaccinated contacts by simulation (Supplementary Figure S1), under the assumption that the direct vaccine efficacy (VE) is 30%, 50% and 70%.

**Figure 4:**
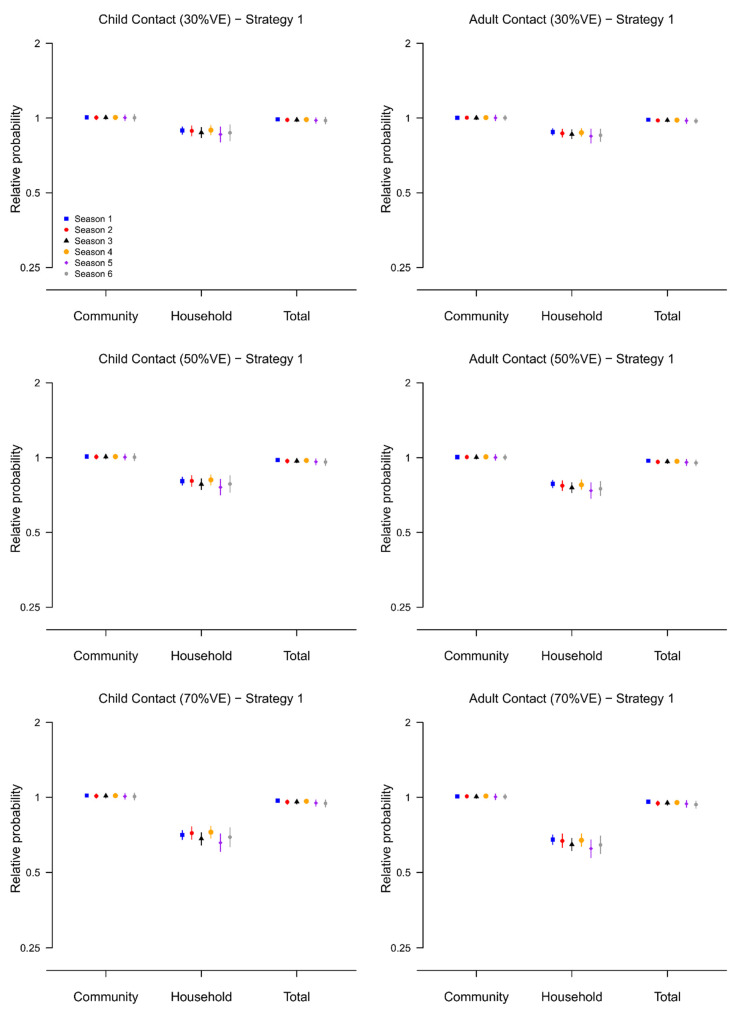
The relative infection probability (from community, from infected household members, or regardless of source) for household contacts of vaccinated children when one child in households is vaccinated (Strategy 2), compared with the scenario when no children in households are vaccinated. Results are presented for the six epidemics, and with assumed VE equal to 30%, 50% and 70%. 95% posterior predictive intervals are constructed with 10,000 simulated epidemics based on the estimated posterior distribution of model parameters (Supplementary Materials).

**Figure 5:**
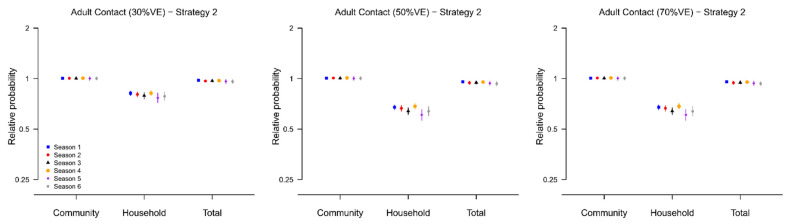
The relative infection probability (from community, from infected household members, or regardless of source) for household contacts of vaccinated children when all children in households are vaccinated (Strategy 2), compared with the scenario when no children in households are vaccinated. Results are presented for the six epidemics, and with assumed VE equal to 30%, 50% and 70%. 95% posterior predictive intervals are constructed with 10,000 simulated epidemics based on the estimated posterior distribution of model parameters (Supplementary Materials).

In the optimal scenario where the direct VE was 70%, we found that, compared to the no vaccination scenario, the probability of household infection for unvaccinated adult contacts was almost halved under both strategies, with relative probabilities ranged from 0.62–0.68 under strategy 1 and 0.44–0.54 under strategy 2 over the six epidemics (Figure 4 and Figure 5). However, the reduction in the total probability of infection was only marginal, with relative probabilities ranged from 0.93–0.96 for strategy 1 and 0.91–0.94 for strategy because the community was by far the main source of infection during the six epidemics in our study (Supplementary Figures S2–S4).

### 3.4. Model Adequacy

We simulated 1000 datasets with parameter values drawn from the posterior distribution. The predicted final size distribution was consistent with the observed data and the model fit was judged adequate (Supplementary Table S2).

### 3.5. Sensitivity Analysis

We refitted the digraph model that relaxed the probabilities of transmission in households, the relative age relative susceptibility and protection from HAI titers were the same (Supplementary Figures S5 and S6). Then, we repeated the simulation to evaluate the degree of indirect protection (Supplementary Figures S7–S11). Similar to the results from main model, we found that even when the vaccine efficacy is 70% and all children are vaccinated, the infection risk of household members of vaccinees could be reduced by 2–14% only. This suggested that our results were robust to this model assumption.

## 4. Discussion

In this study, we estimated the transmission dynamics of influenza A virus in households for six influenza epidemics from 2009–2013 in Hong Kong. We explored factors affecting transmission and evaluate the indirect benefit for their household contacts when vaccinating children in households, for different levels of direct vaccine efficacy.

While vaccination could reduce the probability of transmission in households, its impact on the total probability of infection for household contacts was small. Similar to our previous analysis on influenza B epidemics [16], we estimated that household transmission represented only about 10% of all transmission events in the six influenza A epidemics. This estimate for proportion of household transmission was surprisingly low since other studies that this proportion is 30% [20,21]. The probability of infection from the community could be higher in Hong Kong, due to the crowded public transportation system and schools.

In Hong Kong, the overall vaccine coverage is low. Based on previous studies [34,35,36], the vaccine coverage for children, adults, and the elderly was 18%, 12% and 27% respectively. Therefore, our results should be interpreted as the indirect protection in the household level only. In theory, if the vaccine coverage in a population was high, the probability of infection from the community could decrease due to herd immunity as shown in other studies [13,14,15].

When exposed to a household member with influenza A virus infection, we estimated that the transmission probability was 10% and 14% for children aged <12 and, with a pre-season titer <10. These estimates were similar to those from a case-ascertained study of influenza A virus transmission conducted in Hong Kong [25,34,37]. The probability of transmission within households with smaller number of household members was higher, which was consistent with other studies [20,38,39,40]. These estimates of probabilities of household transmission demonstrated the significance of household transmission when there were infected household members, similar to the estimates based on data from household case-ascertain design [41,42].

We estimated that the susceptibility of infection was decreased with age, which was consistent with estimates obtained for influenza A virus that indicated children are about twice as susceptible as adults, and even more susceptible compared with elderly for pH1N1 but not H3N2 [20,25,37,43]. We estimated that every 2-fold higher in HAI titer was associated with 43% and 28% reduction in infection risk. This was similar to the previous estimates of 50% protection associated with an HAI titer of 40, compared with an HAI titer of <10 [29].

Our study had some limitations. First, we used a 4-fold or greater rise to identify influenza virus infections in our study, which may suffer measurement error for various reasons [44]. Second, between-household transmission was not considered in the analysis. It may be reasonable to assume the households in our studies (~800 households) were independent since they were a small subset of all households in Hong Kong (7M population size).

In conclusion, we estimated the household transmission dynamics in households for six influenza A epidemics and evaluated the potential indirect benefits at the household level of vaccinating children. We found that in an optimistic scenario with 70% VE, vaccinating all children in a household provided a limited (~10%) reduction in infection risk for unvaccinated household members. This suggested that individual vaccination remained important to be protected against influenza.

## Figures and Tables

**Table 1:** Summary of model comparison.

Factors in the Models	∆DIC
Age relative susceptibility + household size + protection of HAI titer	0
Age relative susceptibility + household size + protection of HAI titer + age relative infectivity	30.3
Age relative susceptibility + protection of HAI titer	38.3
Age relative susceptibility + household size	372.9
household size + protection of HAI titer	494.1

## Data Availability

All the data used in the analysis is available at Github: https://github.com/timktsang/influenza_titer_reconstruction (accessed on 15 September 2022).

## Supplementary Materials

The following supporting information can be downloaded at: https://www.mdpi.com/article/10.3390/v14102097/s1, Figure S1: The posterior density of each model parameter in the fitted main model; Figure S2. The probability of infection for child contacts and adult contacts for no vaccination strategy. Point and line indicate the mean and 95% posterior predictive intervals, computed based on 10,000 simulated epidemics; Figure S3. The probability of infection for child contacts and adult contacts for vaccinating one child in each household (Strategy 1). Point and line indicate the mean and 95% posterior predictive intervals, computed based on 10,000 simulated epidemics; Figure S4. The probability of infection for child contacts and adult contacts for vaccinating all children in households (Strategy 2). Point and line indicate the mean and 95% posterior predictive intervals, computed based on 10,000 simulated epidemics; Figure S5. Sensitivity analysis-Probability of infection from community and person-to-person transmission in households for children aged less than 18, with a lower level of titer estimated by the digraph models for 6 epidemics. Point and line are the point estimate and their 95% credible interval; Figure S6. Sensitivity analysis-Estimates of relative susceptibility to infection for age groups and HAI titer. For age group relative susceptibility, the reference group of age is children less than 18 years of age. Panel A: Estimates for age group relative susceptibility. Panel B: Estimates for protection from HAI titer; Figure S7. Sensitivity analysis-The relative infection probability (from community, from infected household members, or regardless of source) for household contacts of vaccinated children when one child in households is vaccinated (Strategy 2), compared with the scenario when no children in households are vaccinated. Results are presented for the six epidemics, and with assumed VE equal to 30%, 50% and 70%. 95% posterior predictive intervals are constructed with 10,000 simulated epidemics based on the estimated posterior distribution of model parameters (Supplementary Methods); Figure S8. Sensitivity analysis-The relative infection probability (from community, from infected household members, or regardless of source) for household contacts of vaccinated children when all children in households are vaccinated (Strategy 2), compared with the scenario when no children in households are vaccinated. Results are presented for the six epidemics, and with assumed VE equal to 30%, 50% and 70%. 95% posterior predictive intervals are constructed with 10,000 simulated epidemics based on the estimated posterior distribution of model parameters (Supplementary Methods); Figure S9. Sensitivity analysis-The probability of infection for child contacts and adult contacts for no vaccination strategy. Point and line indicate the mean and 95% posterior predictive intervals, computed based on 10,000 simulated epidemics (Supplementary Methods); Figure S10. Sensitivity analysis-The probability of infection for child contacts and adult contacts for vaccinating one child in each household (Strategy 1). Point and line indicate the mean and 95% posterior predictive intervals, computed based on 10,000 simulated epidemics (Supplementary Methods); Figure S11. Sensitivity analysis-The probability of infection for child contacts and adult contacts for vaccinating all children in households (Strategy 2). Point and line indicate the mean and 95% posterior predictive intervals, computed based on 10,000 simulated epidemics (Supplementary Methods); Supplementary Table S1. Demographic characteristics of Kiddivax in the 6 influenza A epidemics during study period; Supplementary Table S2. Observed and expected final size distribution in households.
