# Supplementary material for: Indirect Protection from Vaccinating Children against Influenza A Virus Infection in Households
Source: Viruses. 2022 Sep 21;14(10):2097. doi: 10.3390/v14102097 (PMC9610389; doi:10.3390/v14102097)
Supplement: Supplementary file 1 [file viruses-14-02097-s001.zip › viruses-1926877-supplementary.pdf]

Supplementary Information

for 'Indirect protection from vaccinating children against influenza A virus infection in households'

1 SUPPLEMENTARY METHODS 2

1.1 Model for transmission dynamics in households 2

1.1.1 Overview 2

1.1.2 The probability of the digraph 2

1.1.3 Household transmission 3

1.1.4 The probability of infection from the community 4

1.1.5 Susceptibility component 4

1.1.6 Likelihood function for the digraph 4

1.1.7 Agreement between observed data and digraphs 5

1.2 Inference 5

1.2.1 Priors 5

1.2.2 Algorithms 5

1.2.3 Implementation 7

1.3 Model validation 7

1.4 Model Comparison 7

1.5 Model prediction 8

2 SUPPLEMENTARY REFERENCES 9

3 SUPPLEMENTARY FIGURES 11

4 SUPPLEMENTARY TABLES 22

1 SUPPLEMENTARY METHODS

1.1 Model for transmission dynamics in households

For an individual j in household i, we observe a vector $(Y_{ij}, age_{ij}, vac_{ij}, hs_i, AT_{ij})$, where Y_{ij} is the infection status, age_{ij} and vac_{ij} is the age and vaccination status, and hs_i was the household size, AT_{ij} is the log-transformed HAI titer with 0 indicating <10, 1 indicating 10 and so on.

We use a 4-fold or greater rise in consecutive HAI titers to indicate serological evidence of infections. For each individual and each epidemic, the most recent serum specimen collected prior to that epidemic is used to obtain the pre-epidemic HAI titers and the earliest serum after that epidemic is used to obtain the post-epidemic HAI titers. Individuals who were vaccinated as part of the trials, or self-reported receipt of vaccination in any year, are excluded from analyses of that year, since vaccination can also cause 4-fold or greater rise that is indistinguishable from those caused by infections.

To overcome the difficulty that the chains of transmission are not observed, we use a direct graph (digraph) approach to estimate the transmission dynamics in households[1-3] .

1.1.1 Overview

Denote G the digraph indicating the potential transmission chain in households, y the observed data, and θ the parameter vector:

$$P(G, \theta|y) \propto P(y|G)P(G|\theta)P(\theta)$$

Here, $P(y|G)$ is an indicator function equal to 1 if the infection status of all participants derived from the digraph G agrees with the observed infection status y. $P(G|\theta)$ is the probability of digraph G given the parameters θ. $P(\theta)$ is the prior density function of the model parameters θ.

1.1.2 The probability of the digraph

Here, the index for household in the notation are omitted for simplicity. A household of size n is represented by a random directed graph with n vertices, each representing a household member. Edges are added to represent possible transmission events. An

edge between individual *j* and individual *i* indicates that if individual *j* is infected, then individual *i* will be infected. An edge between the community and individual *i* indicates that individual *i* is infected. Those digraphs can be represented by a matrix [1]. In a toy example, where each row represents a potential "source" of transmission while each column represents a potential "recipient":

	Participant 1	Participant 2	Participant 3	Participant 4
Community	0	1	1	0
Participant 1	0	1	0	0
Participant 2	0	0	0	1
Participant 3	1	0	0	0
Participant 4	1	0	0	0

In this digraph, participant 2 and 3 are infected from the community and participant 4 is infected by participant 2. Participant 1 is infected by participant 3.

In this approach, the presence of an edge is independent of that of other edges. Therefore, it is possible to observe both an edge from individual *j* to individual *k* and one edge from individual *k* to individual *j*. For each digraph, we can infer the final infection status for every individual in the household. Denote G_i^j the final outcome for individual *j* in household *i* derived from digraph G_i. The relationship between the digraphs and parameters is summarized in the following sections.

1.1.3 Household transmission

Denote variable v^{jk} the presence of an edge from individual *j* to individual *k*, occurring with the following probability:

$$P\left(v^{jk} = 1 \middle| \theta\right) = 1 - \exp\left(-\lambda^{jk}(\theta)\right)$$

The formulation of $\lambda^{jk}(\theta)$ is as follows:

$$\lambda^{jk}(\theta) = \{\lambda_{h1} I(hs < 4) + \lambda_{h2} I(hs \geq 4)\} * S_k(\theta),$$

where λ_{h1}, λ_{h2} are model parameters for the transmission in households of size < 4 and ≥4, respectively, and $S_k(\theta)$ is the susceptibility variable for individual k described in Section 1.1.5.

1.1.4 The probability of infection from the community

In addition to within household transmission, each of the individual faces a probability of infection from the community. Denote variable v^{Ck} the presence of an edge from the community to individual k, with the following probability

$$P(v^{Ck} = 1|\theta) = 1 - \exp\left(-\lambda^{Ck}(\theta)\right)$$

The formulation of $\lambda^{Ck}(\theta)$ is as follows:

$$\lambda^{Ck}(\theta) = \psi * S_k(\theta)$$

where ψ is a model parameter for infection from the community and $S_k(\theta)$ is the susceptibility component for individual k described in section 1.2.3.

1.1.5 Susceptibility component

For an individual k, his/her susceptibility is:

$$S_k = \exp\{\beta_1 \mathrm{I}(age_k \in [12{,}17]) + \beta_2 \mathrm{I}(age_k \in [18{,}44]) + \beta_3 \mathrm{I}(age_k \in [45{,}64]) + \beta_4 \mathrm{I}(age_k \geq 65) + \beta_5 AT_k\},$$

where $\exp(\beta_1)$, $\exp(\beta_2)$, $\exp(\beta_3)$ and $\exp(\beta_4)$ are the age group relative susceptibility compared with children aged less than 12. $\exp(\beta_5)$ is the relative susceptibility associated with 2-fold higher HAI titers. We also separate all parameters for H1N1 and H3N2. Susceptibility is defined on be positive, from 0 to infinity. For the reference group, the relative susceptibility is 1. If relative susceptibility > 1 for an individual, then s/he has a higher infection risk compared with the individuals in reference group.

1.1.6 Likelihood function for the digraph

For a given household, the likelihood contribution for the digraph is:

$$L(G|\theta) = \prod_{j:j=0|y_j=1} \prod_{k \neq j} P\left(v^{jk} = 1\middle|\theta\right)^{v^{jk}} P\left(v^{jk} = 0\middle|\theta\right)^{1-v^{jk}}$$

Note that j is started from 0 to represent the infection from the community.

We assume households are independent of each other so that the full likelihood is the product of all household likelihoods.

1.1.7 Agreement between observed data and digraphs

The second level of the model ensure that the proposed digraph, and hence the potential transmission chain, agrees with the observed data:

$$P(y|G) = \prod_i \prod_j I(G_i^j = y_{ij})$$

where G_i^j the final outcome for individual j in household i derived from digraph G_i.

1.2 Inference

We use a data augmentation MCMC approach to explore the parameters and digraph space [2], to estimate the posterior distribution of model parameters. We outline the algorithm in the following sections.

1.2.1 Priors

For parameters describing the strength of infection in the community ψ and the strength of transmission in households $\lambda_{h1}, \lambda_{h2}$, the prior distribution is a Uniform(0,10) distribution. Then, (2.5%,97.5%) percentile of the probability of transmission, that is equal to $1 - \exp(-h)$, where h could be ψ, λ_{h1} or λ_{h2}, is (0, 0.9999).

For those parameters that related to the susceptibility, the priors are Normal(0,3). The (2.5%,97.5%) percentile of the exponential of this prior would be (0.003, 358).

1.2.2 Algorithms

At the initial step, we do the following.

Because there are a small number of missing values for pre-season titers, we first impute the missing values in antibody titers level by using their observed empirical distribution. The distributions could be different for children and adults.

As explained in previous studies [1, 2], the data augmentation approach could be restricted to edges between participants that might potentially have been infected (i.e.

with final outcome being infected or unknown). We define potential edges as edges between participants that might have been infected. We define a non-edge as the absence of a potential edge.

We start from a full digraph, assuming all potential edges in the digraph are present and hence all participants with unknown infection status are infected and updated the digraph and the unknown status in the MCMC algorithm.

At each MCMC step, we do the following updates:

For the model parameter vector θ, we use a metropolis-hasting algorithm to update each of the parameters individually.

We update the digraph G and the unknown infection status by first deciding to add a potential edge or delete an edge with equal probability.

To add an edge, we randomly select a non-edge from all the non-edges (including both household and community edges). Next, we compute the corresponding digraph and infection status for participants with unknown infection status. No further checking is needed, since adding an edge would not change the consistency between digraphs and observed data.

Suppose the total number of potential edges is A and the number of edges in this step is B. Then the probability that accepting the addition of this edge would be

$$\min\left(1, \frac{L\left(G' \middle| \theta\right)}{L(G|\theta)} * \frac{\frac{1}{B+1}}{\frac{1}{A-B}}\right)$$

where $L(G'|\theta)/L(G|\theta)$ was the likelihood ratio of the current digraph G (without the proposed edge) and the proposed digraph G' (with the proposed edge and updated unknown infection status).

For deleting an existing edge, we need to ensure the digraph would be consistent with the observed data, so that every confirmed infection in the observed data set should have at least one edge from the community or other infected household members.

Otherwise, the deletion would be directly rejected. After checking the consistency, the acceptance probability for the deletion is:

$$\min\left(1, \frac{L\left(G^{'}\middle|\theta\right)}{L(G|\theta)} * \frac{\frac{1}{A-B+1}}{\frac{1}{B}}\right)$$

where G' was the digraph with the proposed deletion and updated unknown infection status and G was the digraph without the proposed deletion. Re-computing the infection status in the selected household is necessary because deleting an edge would change the infection status of household members with unknown infection status.

After updating the digraph, we also update the missing antibody titers level by using metropolis-hasting algorithm.

1.2.3 Implementation

The chain was run for 200,000 iterations with a burn-in of 100,000 and a thinning of 10. The algorithm is implemented in R with Rcpp package so that C++ could be used. One run of the algorithm for 200000 iterations took about 120 minutes on a desktop with processor: Inter® Xeon® CPU W3565 @3.20GHz.

1.3 Model validation

We use the best fitted model to predict the final size distribution and summarized in Supplementary Table 2. All credible intervals can cover the observed number of infections, suggesting that the model fit is adequate.

1.4 Model Comparison

The likelihood of the observed data is not available in this approach, therefore it is estimated by an importance sampling method [1, 4]. For each household, 2000 datasets are simulated, with parameters drawn from the posterior distribution. Then the observed data and simulated data are compared. The likelihood contribution of a household is equal to the proportion of simulated data with infection status that exactly matched the observed data, for all household members. To avoid the problem of 0-valued likelihood, we use the approach developed by Cauchemez et al[1], with assuming the sensitivity and specificity for diagnosing a case were both 99.99%.

After using the above-mentioned approach to estimate the likelihood of the observed data, the DIC is computed as $2\overline{D} - D(\bar{\theta})$, where D is the deviance, equal to -2*log of likelihood.

1.5 Model prediction

To evaluate indirect protection, a simulation study is conducted, with parameters drawn from the posterior distribution. Two vaccine strategies are evaluated:

- Strategy 1: vaccinating one child in each household;
- Strategy 2: vaccinating all children in each household.

and compared to the strategy of “no vaccination”.

10,000 epidemics in 150000 households are simulated with parameters drawn from their posterior distribution. The structure of a simulated household is identical to that of a household randomly drawn in the study. This refers only to the household size and demographics, but not to the directed edges between household members, and that a digraph was then constructed for each household in each of the simulated epidemics. Then, we also add the vaccinated individual's susceptibility S_k set to a fixed value, representing the chosen VE. For example, if VE is 70%, then relative susceptibility is 0.3, so that multiple S_k by 0.3 for those individual.

For each infected individual, the source of infection for this individual could be determined based on the recorded digraph with the following algorithm:

1) If the individual only has edges from the community only, the source of infection is the community.

2) Else, if the individual only has edges from other infected household members, the source of infection is the household.

3) Else, if the individual is the only one with an edge from the community, the source of infection is the community.

4) Otherwise, the source of infection is inconclusive. In our analysis, half of infections with inconclusive source are assigned to the community and the other half to households.

For each strategy, we can compute a probability of infection for a given group and from a specific source (Supplementary Figures 1,2). For a given group (children or adults) and a given source of infection (household, community or both), we measure the indirect protection due to a vaccine strategy by the ratio of probability of infection in that group and from that source under this vaccine strategy, compared to the probability of infection under no vaccination strategy.

These simulations are repeated for each parameter vector randomly drawn from posterior, hence 95% posterior predictive intervals are derived for each of these relative probabilities that correctly captured the effect of parameter uncertainty on model predictions. These relative probabilities is recomputed using 100000 households and they were basically the same.

2 Sensitivity analysis

In the main analysis, we only allowed the probabilities of infection from community could vary by epidemics (6 parameters), while all other parameters, including probabilities of person-to-person transmission in households, the age relative susceptibility, and the protection from HAI titers, were assumed to depend on subtype only (but could not vary by epidemics).

While these parameters should not be epidemic-dependent, since 1) age relative susceptibility and protection from HAI titers were biological factors that should not vary by epidemics, 2) the probabilities of person-to-person transmission was shown to be stable, when there was infected household members based on previous household case-ascertain studies [1,3].

To determine if our conclusion that the degree of indirect protection was small is robust to this assumption, we fit the following model that allow for epidemic-specific parameters for these parameters. For age relative susceptibility, we have to modify it to children (<18) and adults (18+) only, since the sample size is not sufficient.

3 SUPPLEMENTARY REFERENCES

1. Cauchemez S, Ferguson NM, Fox A, Mai le Q, Thanh le T, Thai PQ, et al. Determinants of influenza transmission in South East Asia: insights from a household cohort study in Vietnam. PLoS Pathog. 2014;10(8):e1004310. doi:

10.1371/journal.ppat.1004310. PubMed PMID: 25144780; PubMed Central PMCID: PMC4140851.
2. Demiris N, O'Neill PD. Bayesian inference for stochastic multitype epidemics in structured populations via random graphs. Journal of the royal Statistical Society Series B. 2005;67:731-45.
3. Tsang TK, Fang VJ, Ip DKM, Perera R, So HC, Leung GM, et al. Indirect protection from vaccinating children against influenza in households. Nat Commun. 2019;10(1):106. Epub 2019/01/12. doi: 10.1038/s41467-018-08036-6. PubMed PMID: 30631062; PubMed Central PMCID: PMCPMC6328591.
4. Liu JS. Monte Carlo strategies in scientific computing. New York: Springer-Verlag; 2001.

4 SUPPLEMENTARY FIGURES

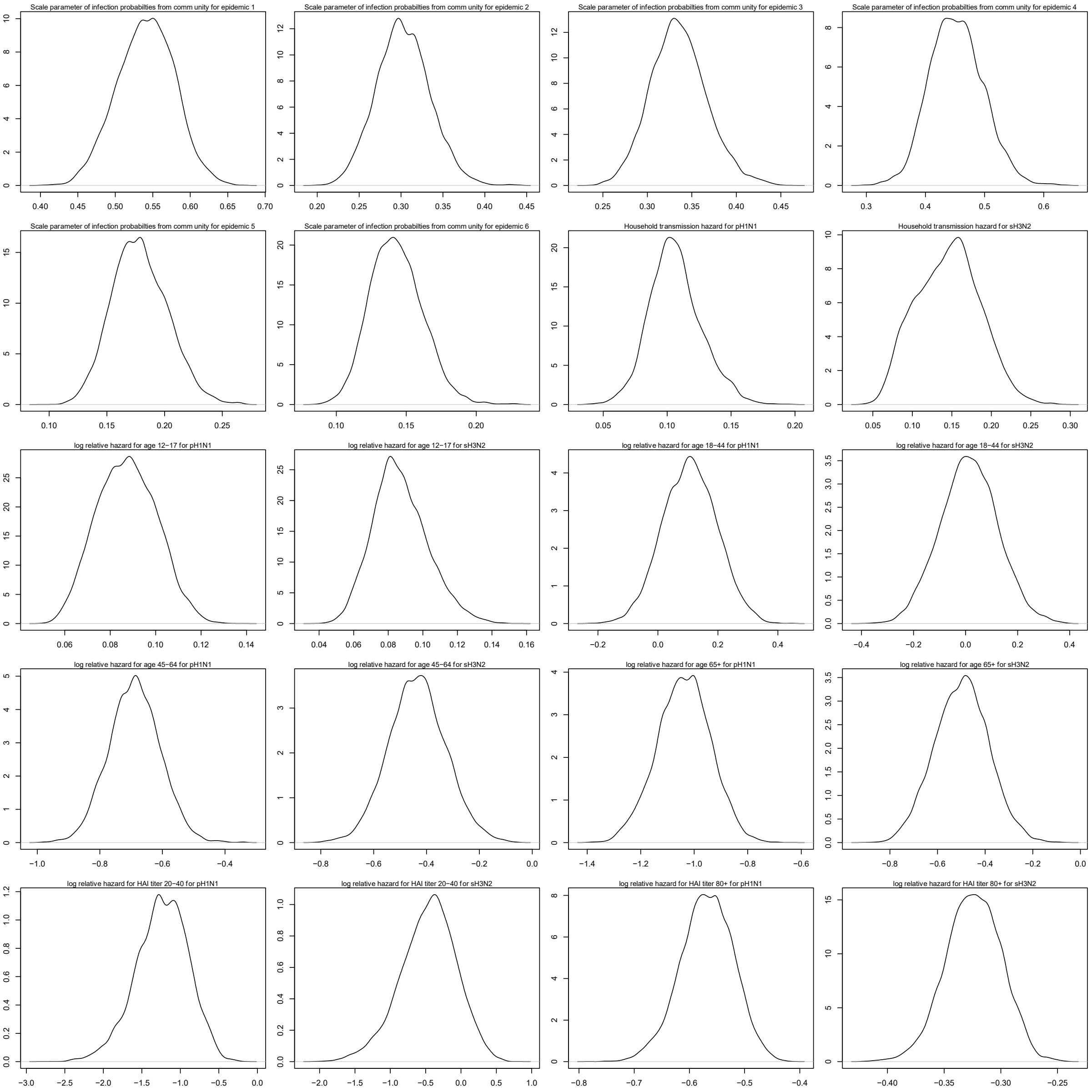

Supplementary Figure S1. The posterior density of each model parameter in the fitted main model.

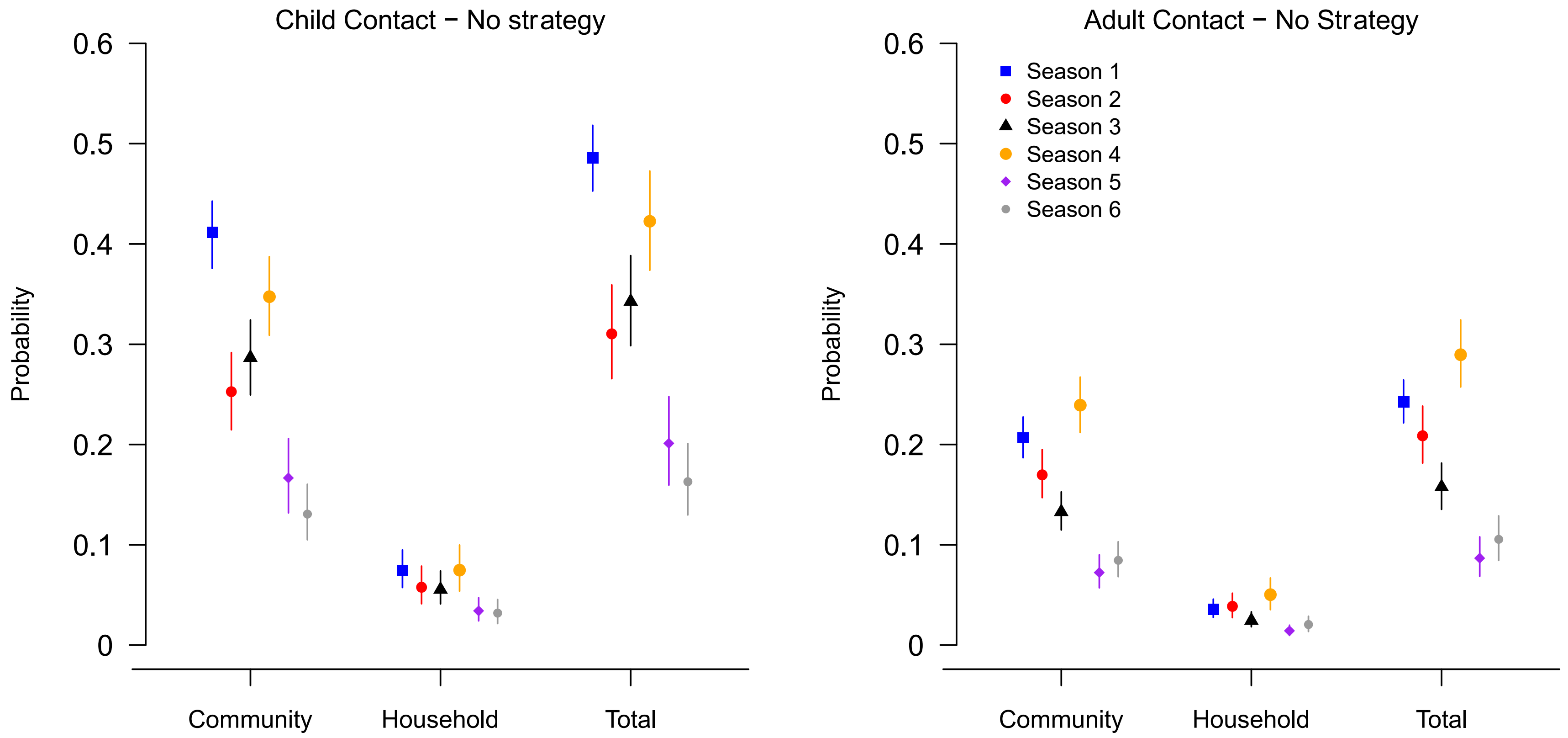

Supplementary Figure S2. The probability of infection for child contacts and adult contacts for no vaccination strategy. Point and line indicate the mean and 95% posterior predictive intervals, computed based on 10000 simulated epidemics (Supplementary Methods).

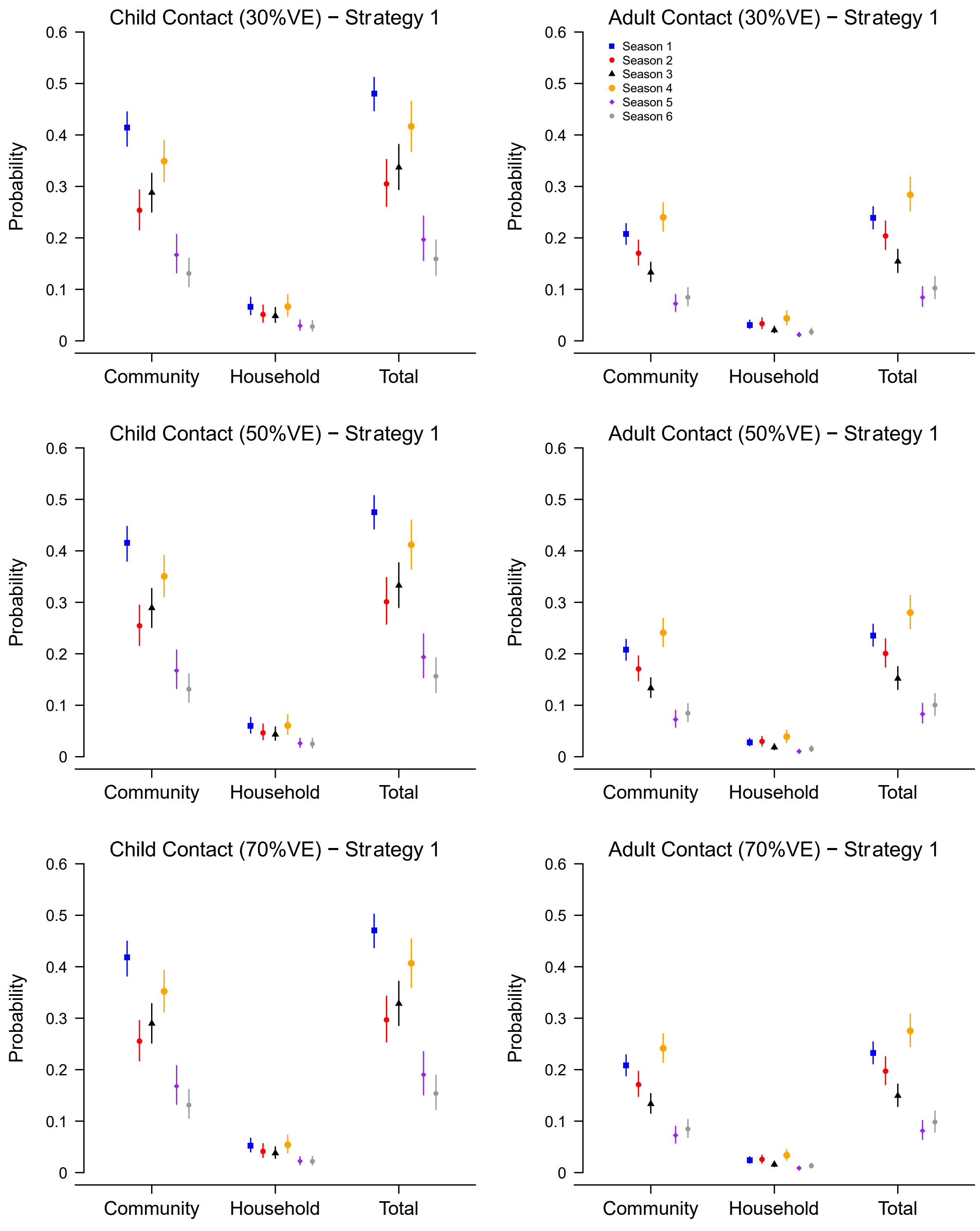

Supplementary Figure S3. The probability of infection for child contacts and adult contacts for vaccinating one child in each household (Strategy 1). Point and line indicate the mean and 95% posterior predictive intervals, computed based on 10000 simulated epidemics (Supplementary Methods).

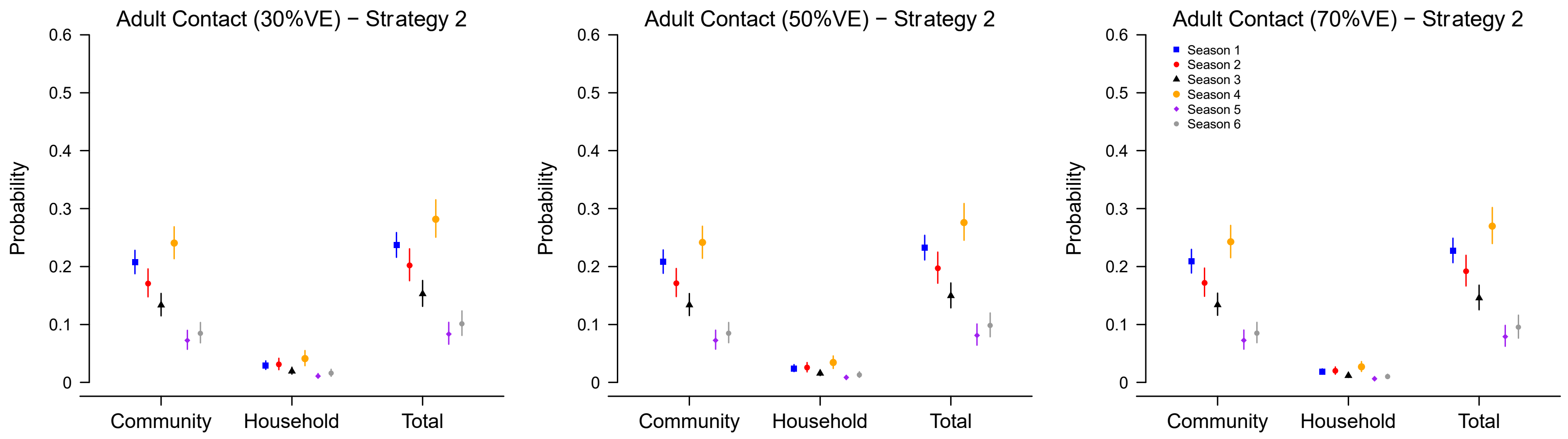

Supplementary Figure S4. The probability of infection for child contacts and adult contacts for vaccinating all children in households (Strategy 2). Point and line indicate the mean and 95% posterior predictive intervals, computed based on 10000 simulated epidemics (Supplementary Methods).

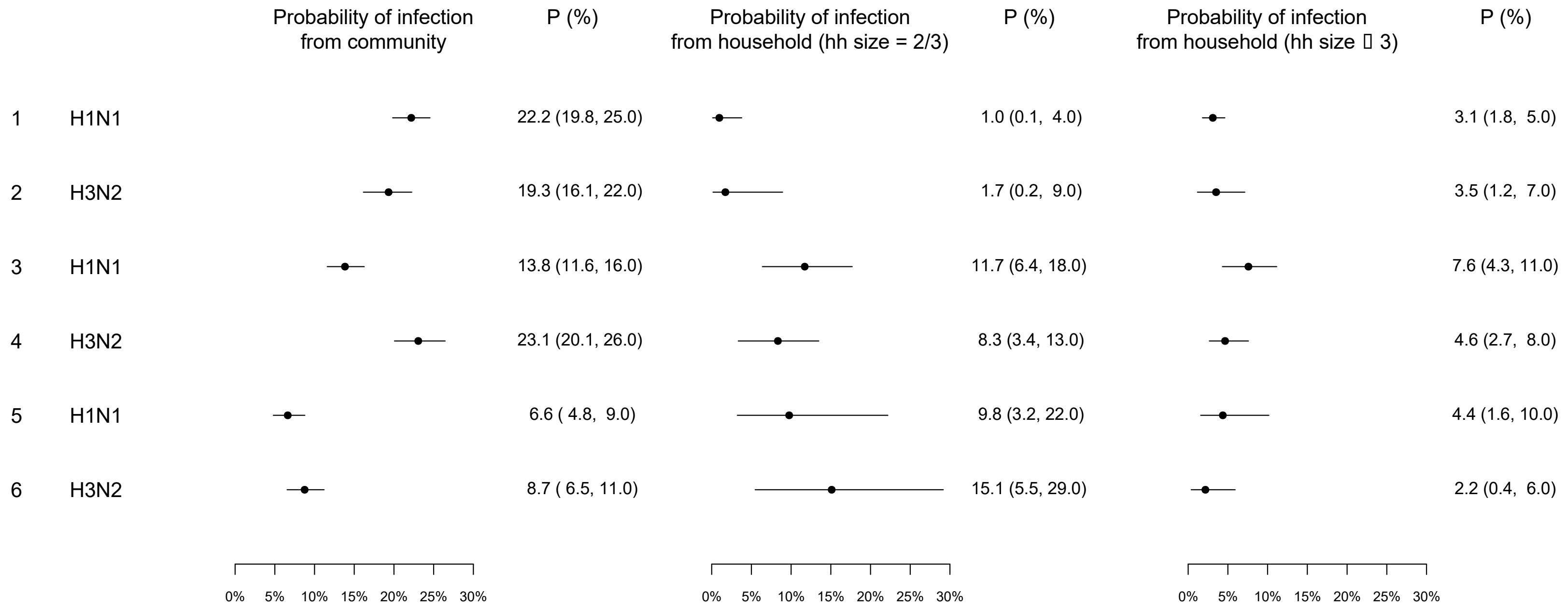

Supplementary Figure S5. Sensitivity analysis - Probability of infection from community and person-to-person transmission in households for children aged less than 18, with a lower level of titer estimated by the digraph models for 6 epidemics. Point and line are the point estimate and their 95% credible interval.

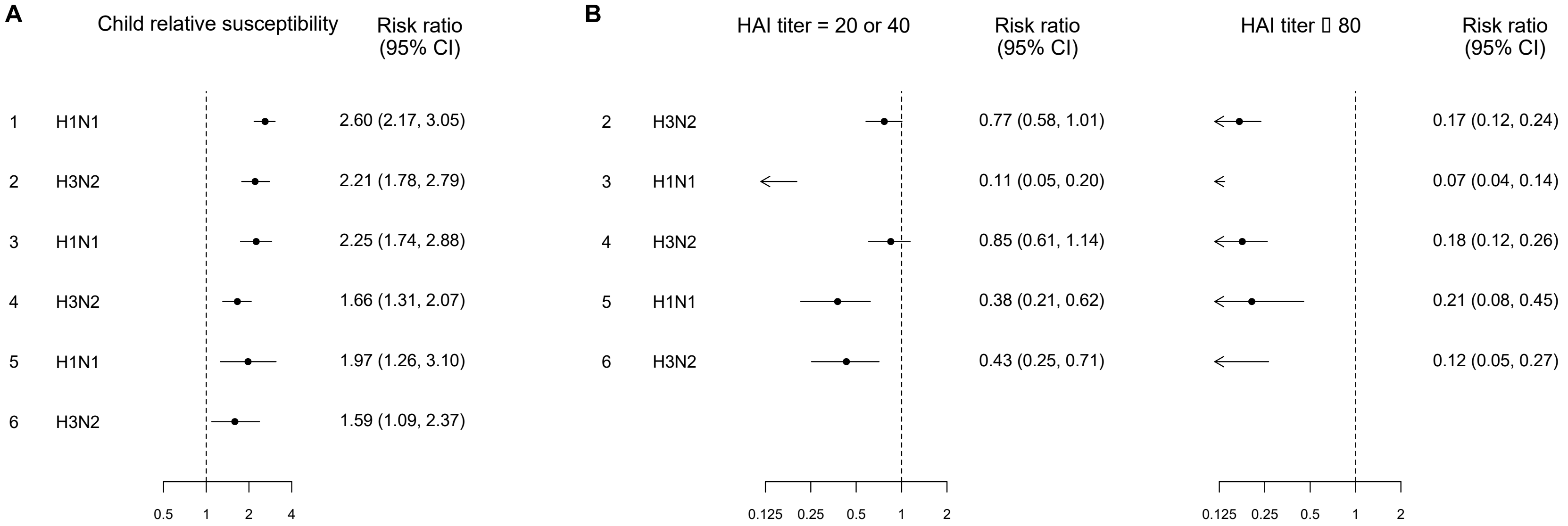

Supplementary Figure S6. Sensitivity analysis - Estimates of relative susceptibility to infection for age groups and HAI titer. For age group relative susceptibility, the reference group of age is children less than 18 years of age. Panel A: Estimates for age group relative susceptibility. Panel B: Estimates for protection from HAI titer.

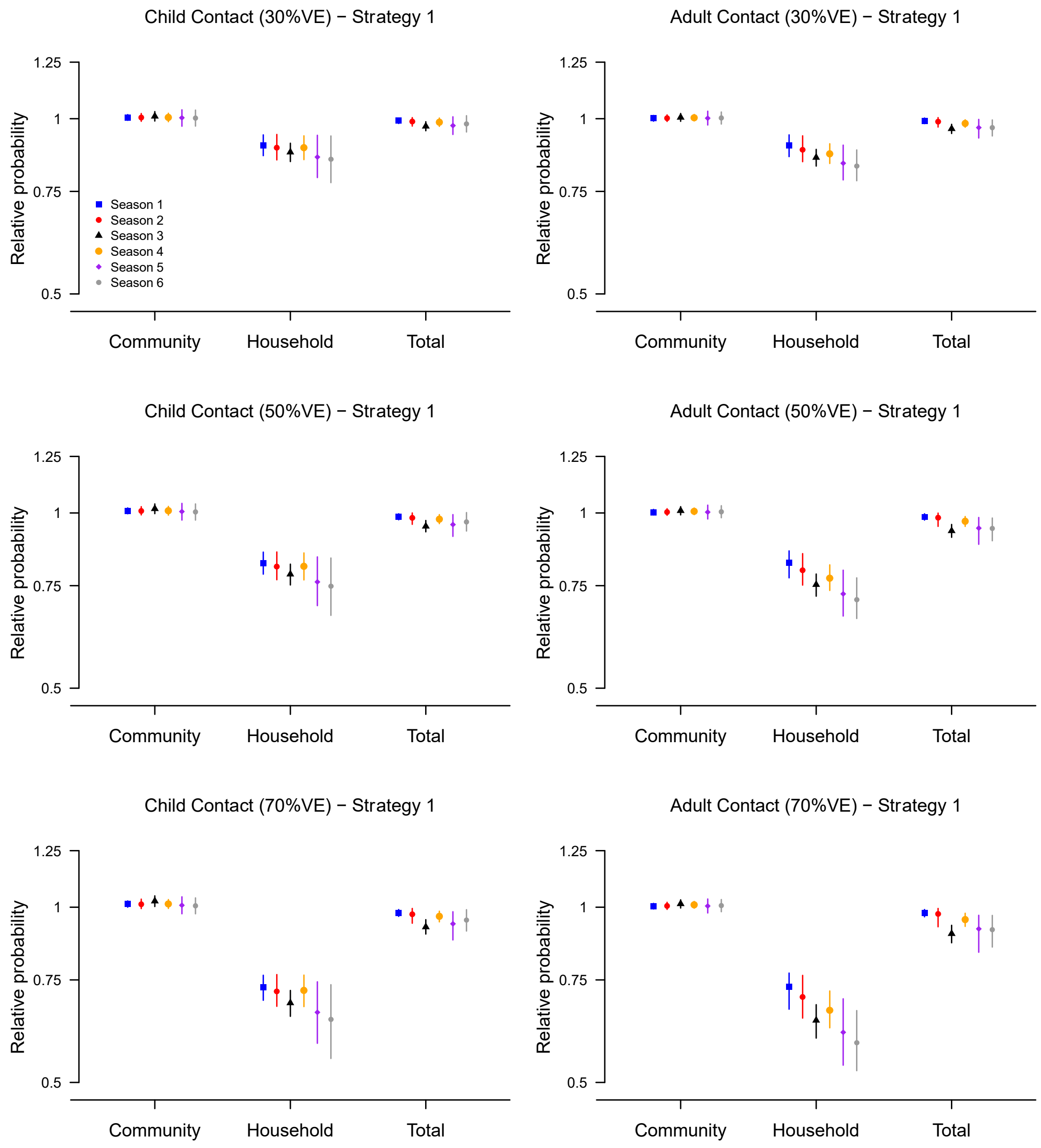

Supplementary Figure S7. Sensitivity analysis - The relative infection probability (from community, from infected household members, or regardless of source) for household contacts of vaccinated children when one child in households is vaccinated (Strategy 2), compared with the scenario when no children in households are vaccinated. Results are presented for the six epidemics, and with assumed VE equal to 30%, 50% and 70%. 95% posterior predictive intervals are constructed with 10000 simulated epidemics based on the estimated posterior distribution of model parameters (Supplementary Methods).

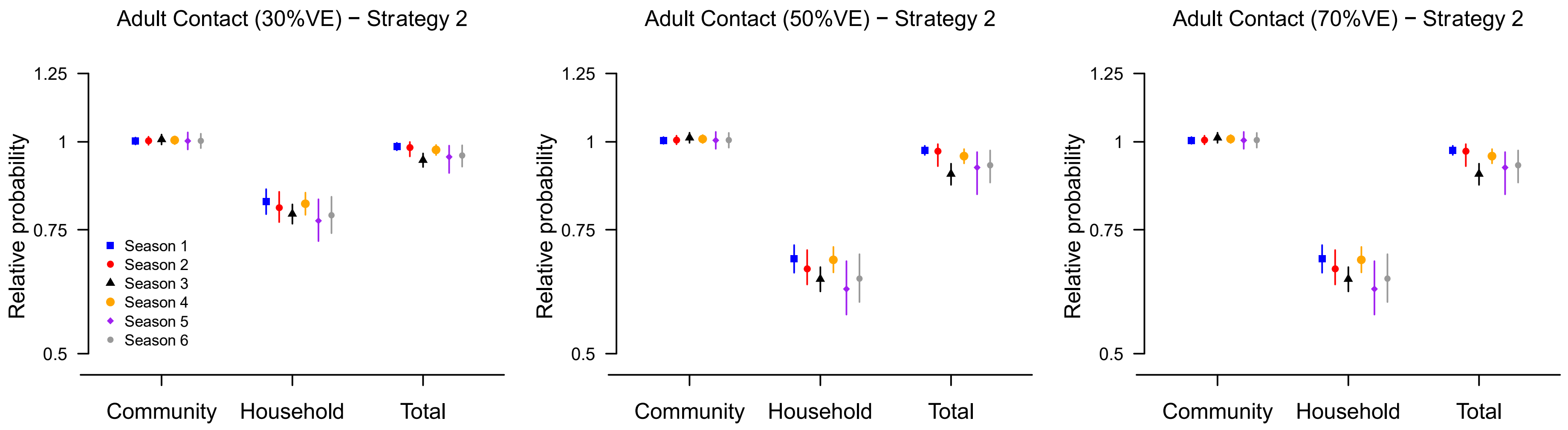

Supplementary Figure S8. Sensitivity analysis - The relative infection probability (from community, from infected household members, or regardless of source) for household contacts of vaccinated children when all children in households are vaccinated (Strategy 2), compared with the scenario when no children in households are vaccinated. Results are presented for the six epidemics, and with assumed VE equal to 30%, 50% and 70%. 95% posterior predictive intervals are constructed with 10000 simulated epidemics based on the estimated posterior distribution of model parameters (Supplementary Methods).

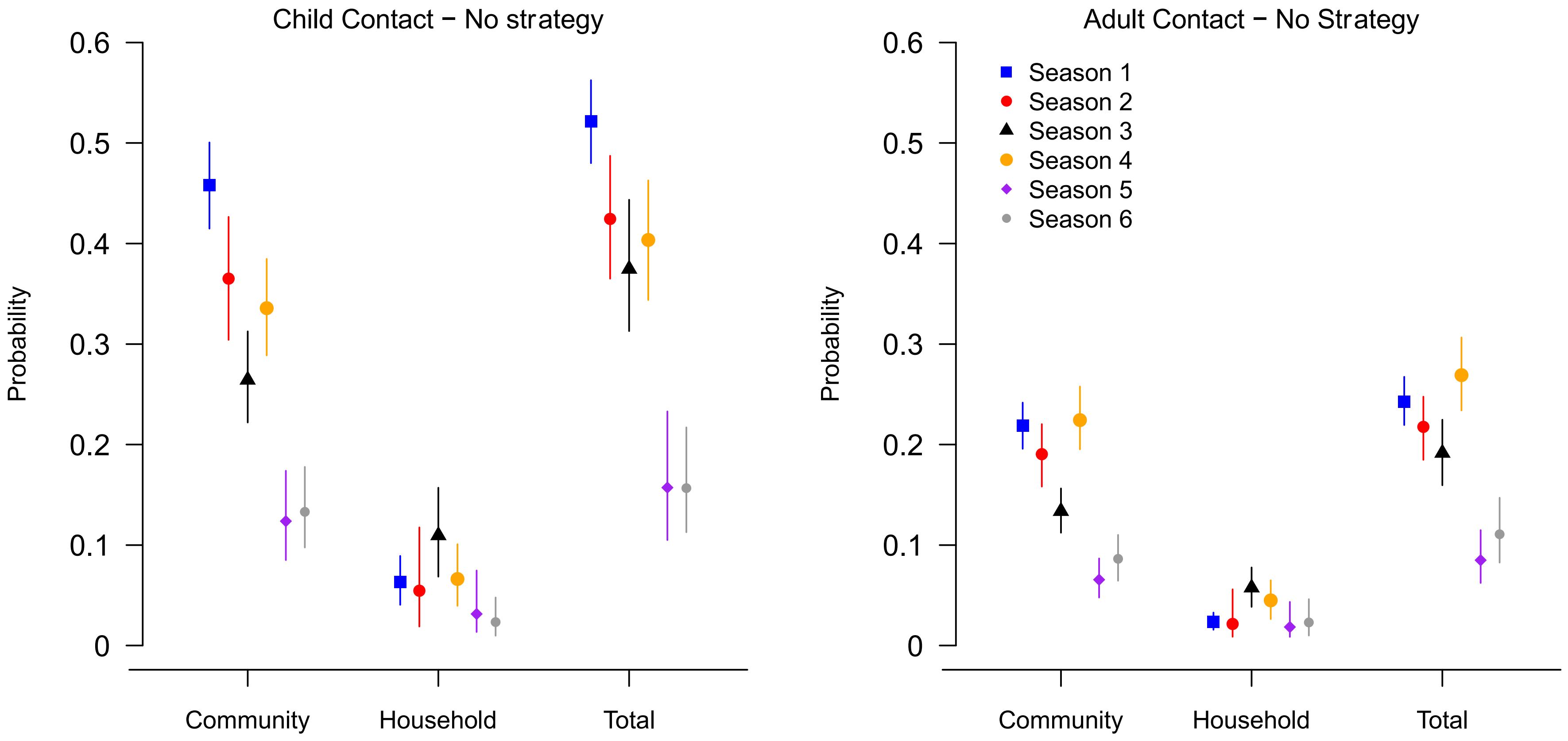

Supplementary Figure S9. Sensitivity analysis - The probability of infection for child contacts and adult contacts for no vaccination strategy. Point and line indicate the mean and 95% posterior predictive intervals, computed based on 10000 simulated epidemics (Supplementary Methods).

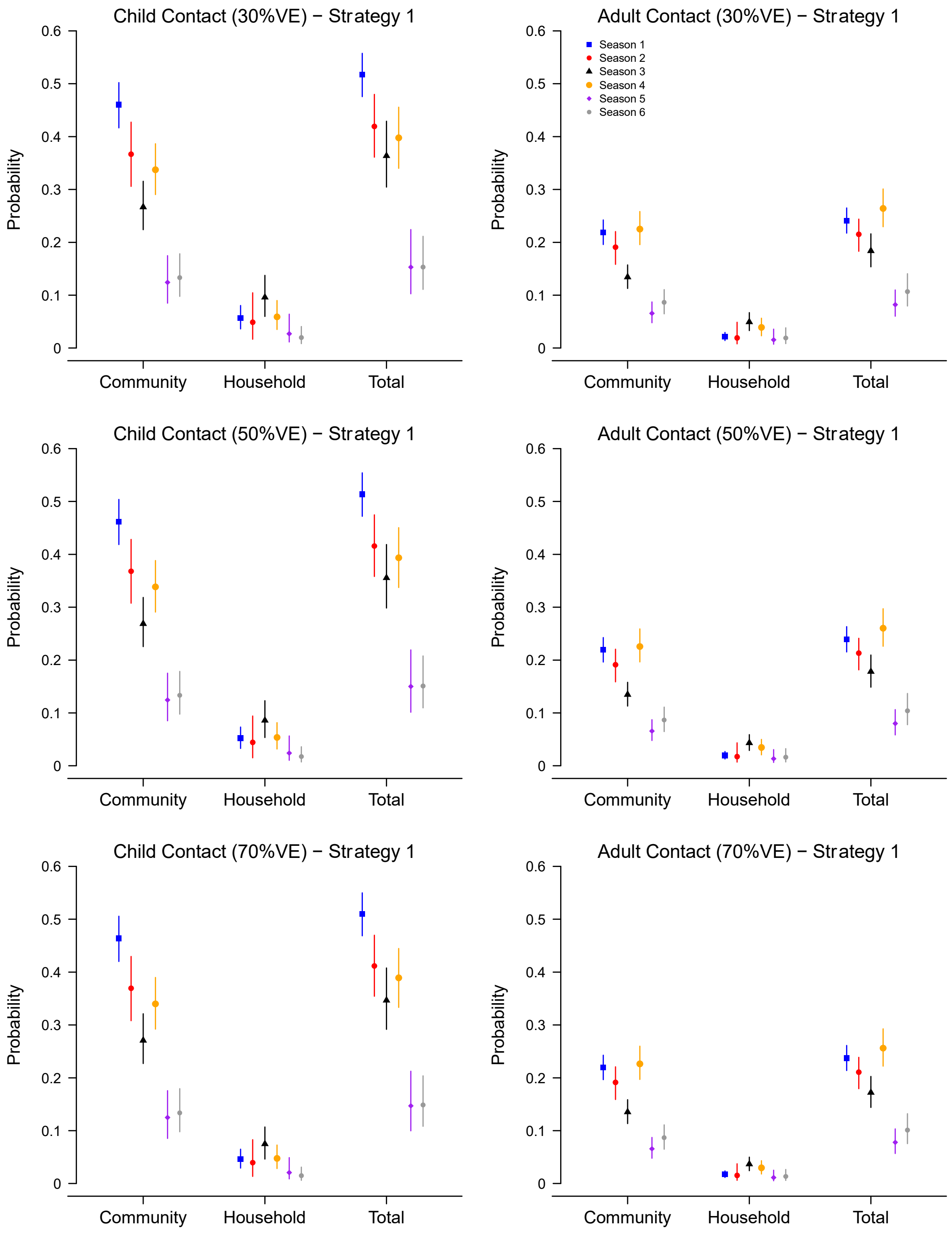

Supplementary Figure S10. Sensitivity analysis - The probability of infection for child contacts and adult contacts for vaccinating one child in each household (Strategy 1). Point and line indicate the mean and 95% posterior predictive intervals, computed based on 10000 simulated epidemics (Supplementary Methods).

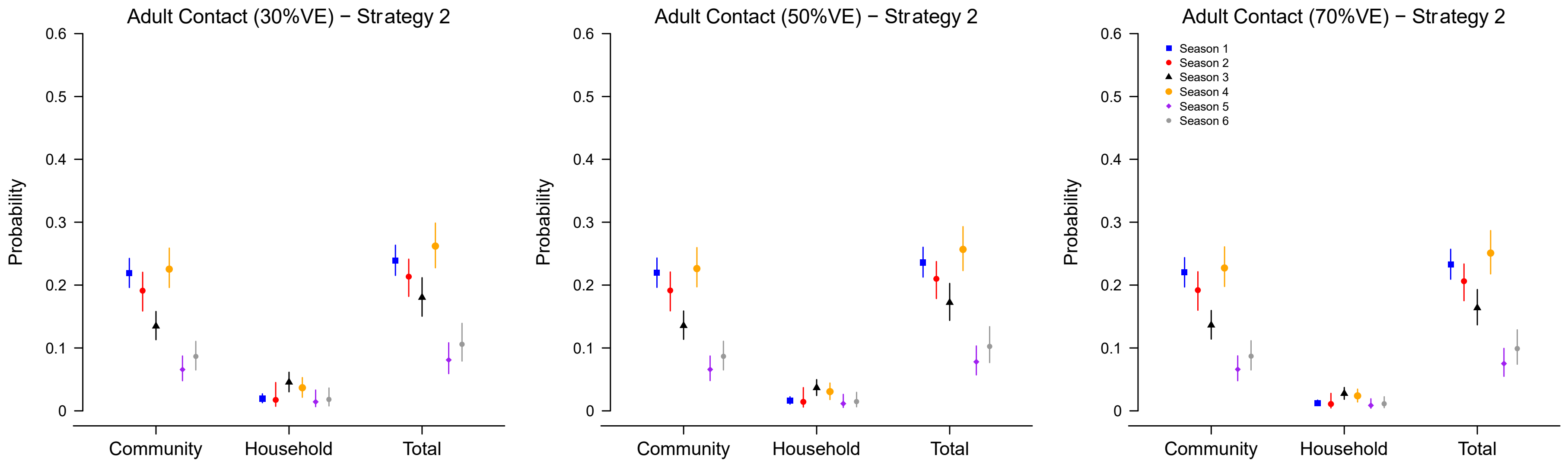

Supplementary Figure S11. Sensitivity analysis - The probability of infection for child contacts and adult contacts for vaccinating all children in households (Strategy 2). Point and line indicate the mean and 95% posterior predictive intervals, computed based on 10000 simulated epidemics (Supplementary Methods).

5 SUPPLEMENTARY TABLES

Supplementary Table S1. Demographic characteristics of Kiddivax in the 6 influenza A epidemics during study period.

	**1: pH1N1** N=2512	**2: H3N2** N=1943	**3: pH1N1** N=1929	**4: H3N2** N=1536	**5: pH1N1** N=1444	**6: H3N2** N=1443	**Overall** N=10807
**Age**							
Children (0-17)	1076/2451 (43.9 %)	845/1894 (44.6 %)	820/1879 (43.6 %)	609/1425 (42.7 %)	566/1323 (42.8 %)	562/1318 (42.6 %)	4478/10290 (43.5 %)
Adults (≥18)	1375/2451 (56.1 %)	1049/1894 (55.4 %)	1059/1879 (56.4 %)	816/1425 (57.3 %)	757/1323 (57.2 %)	756/1318 (57.4 %)	5812/10290 (56.5 %)
**Sex**							
Female	1319/2448 (53.9 %)	1010/1891 (53.4 %)	1001/1876 (53.4 %)	754/1422 (53 %)	707/1320 (53.6 %)	705/1315 (53.6 %)	5496/10272 (53.5 %)
Male	1129/2448 (46.1 %)	881/1891	875/1876	668/1422	613/1320	610/1315	4776/10272 (46.5 %)

		(46.6 %)	(46.6 %)	(47 %)	(46.4 %)	(46.4 %)	
**HAI titer**							
0 - 1	2455/2455 (100 %)	1051/1886 (55.7 %)	1152/1867 (61.7 %)	875/1408 (62.1 %)	697/1297 (53.7 %)	645/1305 (49.4 %)	6875/10218 (67.3 %)
2 - 3	0/2455 (0 %)	231/1886 (12.2 %)	307/1867 (16.4 %)	168/1408 (11.9 %)	404/1297 (31.1 %)	372/1305 (28.5 %)	1482/10218 (14.5 %)
≥ 4	0/2455 (0 %)	604/1886 (32 %)	408/1867 (21.9 %)	365/1408 (25.9 %)	196/1297 (15.1 %)	288/1305 (22.1 %)	1861/10218 (18.2 %)
**Infection status**							
Children	381/612 (62.3 %)	84/476 (17.6 %)	118/755 (15.6 %)	166/604 (27.5 %)	46/561 (8.2 %)	47/558 (8.4 %)	842/3566 (23.6 %)
Adults	314/1239 (25.3 %)	173/951 (18.2 %)	128/985 (13 %)	178/804 (22.1 %)	45/736 (6.1 %)	54/746 (7.2 %)	892/5461 (16.3%)
Overall	695/1851 (37.5 %)	257/1427 (18 %)	246/1740 (14.1 %)	344/1408 (24.4 %)	91/1297 (7 %)	101/1304 (7.7 %)	1734/9027 (19.2 %)

Supplementary Table S2. Observed and expected final size distribution in households

		Numbers of infections							
		0	1	2	3	4	5	6	7
		Epidemics 1							
Numbers of household member	2	11 - 14.58 (9, 20)	15 - 15.77 (10, 21)	8 - 3.66 (1, 8)	NA	NA	NA	NA	NA
	3	108 - 115.77 (99, 133)	103 - 105.8 (90, 122)	44 - 33.72 (23, 46)	4 - 3.71 (0, 8)	NA	NA	NA	NA
	4	44 - 51.16 (38, 65)	77 - 77.64 (64, 91)	55 - 50.26 (38, 63)	18 - 15.94 (9, 24)	3 - 1.99 (0, 5)	NA	NA	NA
	5	8 - 11.5 (6, 18)	15 - 19.58 (13, 27)	20 - 16.15 (10, 23)	11 - 7.65 (3, 13)	3 - 1.92 (0, 5)	0 - 0.2 (0, 1)	NA	NA
	6	3 - 2.18 (0, 5)	4 - 3.83 (1, 7)	3 - 3.51 (1, 7)	2 - 2.13 (0, 5)	0 - 0.96 (0, 3)	1 - 0.33 (0, 2)	0 - 0.06 (0, 1)	NA
	7	0 - 0.08 (0, 1)	0 - 0.26 (0, 1)	0 - 0.49 (0, 2)	1 - 0.55 (0, 2)	0 - 0.41 (0, 2)	1 - 0.16 (0, 1)	0 - 0.05 (0, 1)	0 - 0 (0, 0)
		Epidemics 2							
Numbers of household contacts	2	40 - 44.84 (37, 52)	22 - 19.41 (12, 27)	5 - 2.74 (0, 6)	NA	NA	NA	NA	NA
	3	144 - 138.71 (121, 157)	81 - 80.26 (62, 97)	15 - 19.9 (11, 29)	1 - 2.14 (0, 6)	NA	NA	NA	NA
	4	76 - 79.23 (65, 95)	61 - 62.96 (48, 77)	32 - 27.06 (18, 37)	7 - 6.88 (2, 13)	1 - 0.86 (0, 3)	NA	NA	NA
	5	16 - 18.76 (12, 25)	22 - 15.92 (10, 22)	2 - 7.54 (3, 13)	5 - 2.32 (0, 6)	0 - 0.42 (0, 2)	0 - 0.04 (0, 1)	NA	NA

	6	2 - 2.33 (0, 5)	3 - 2.76 (0, 6)	2 - 1.82 (0, 4)	0 - 0.81 (0, 3)	1 - 0.23 (0, 1)	0 - 0.04 (0, 1)	0 - 0 (0, 0)	NA
	7	0 - 0.13 (0, 1)	0 - 0.23 (0, 1)	0 - 0.26 (0, 1)	1 - 0.21 (0, 1)	0 - 0.12 (0, 1)	0 - 0.04 (0, 1)	0 - 0.01 (0, 0)	0 - 0 (0, 0)
		Epidemics 3							
Numbers of household contacts	2	49 - 49.46 (43, 56)	13 - 12.27 (6, 19)	2 - 2.27 (0, 6)	NA	NA	NA	NA	NA
	3	170 - 166.88 (151, 182)	55 - 52.83 (39, 67)	9 - 16.17 (8, 25)	5 - 3.12 (0, 8)	NA	NA	NA	NA
	4	99 - 105.98 (92, 120)	54 - 46.75 (34, 60)	16 - 17.06 (9, 25)	5 - 4.53 (1, 10)	1 - 0.68 (0, 3)	NA	NA	NA
	5	25 - 25.12 (19, 31)	12 - 11.76 (6, 18)	7 - 4.91 (1, 9)	0 - 1.69 (0, 5)	0 - 0.46 (0, 2)	0 - 0.07 (0, 1)	NA	NA
	6	5 - 3.79 (1, 6)	1 - 2.25 (0, 5)	0 - 1.2 (0, 3)	1 - 0.57 (0, 2)	1 - 0.17 (0, 1)	0 - 0.02 (0, 0)	0 - 0 (0, 0)	NA
	7	0 - 0.54 (0, 1)	0 - 0.31 (0, 1)	1 - 0.12 (0, 1)	0 - 0.03 (0, 1)	0 - 0.01 (0, 0)	0 - 0 (0, 0)	0 - 0 (0, 0)	0 - 0 (0, 0)
		Epidemics 4							
Numbers of household contacts	2	43 - 42.79 (35, 51)	19 - 20.84 (13, 28)	6 - 4.37 (1, 9)	NA	NA	NA	NA	NA
	3	99 - 105.6 (90, 121)	69 - 67.64 (53, 83)	32 - 25.39 (16, 36)	4 - 5.37 (1, 11)	NA	NA	NA	NA
	4	63 - 59.47 (47, 72)	46 - 51.77 (40, 63)	30 - 24.62 (16, 34)	3 - 7.13 (2, 13)	2 - 1.01 (0, 3)	NA	NA	NA
	5	12 - 12.34 (7, 18)	12 - 11.63 (6, 17)	5 - 6.71 (3, 11)	5 - 2.6 (0, 6)	0 - 0.64 (0, 2)	0 - 0.07 (0, 1)	NA	NA
	6	0 - 1.28 (0, 3)	3 - 1.45 (0, 4)	2 - 1.12 (0, 3)	0 - 0.72 (0, 2)	0 - 0.32 (0, 2)	0 - 0.1 (0, 1)	0 - 0.02 (0, 0)	NA
		Epidemics 5							
	2	52 - 53.14 (48, 58)	7 - 6.06 (2, 11)	1 - 0.8 (0, 3)	NA	NA	NA	NA	NA

Numbers of household contacts	3	168 - 163.4 (151, 174)	20 - 23.57 (13, 35)	5 - 5.16 (1, 11)	0 - 0.87 (0, 4)	NA	NA	NA	NA
	4	101 - 106.64 (96, 117)	28 - 22.17 (13, 32)	2 - 4.4 (1, 9)	3 - 0.72 (0, 3)	0 - 0.07 (0, 1)	NA	NA	NA
	5	25 - 24.38 (19, 29)	5 - 5.81 (2, 11)	2 - 1.42 (0, 4)	0 - 0.32 (0, 2)	0 - 0.06 (0, 1)	0 - 0.01 (0, 0)	NA	NA
	6	4 - 3.62 (2, 5)	1 - 0.98 (0, 3)	0 - 0.29 (0, 2)	0 - 0.07 (0, 1)	0 - 0.02 (0, 0)	0 - 0 (0, 0)	0 - 0 (0, 0)	NA
		Epidemics 6							
Numbers of household contacts	2	54 - 52.73 (47, 57)	5 - 6.31 (2, 11)	1 - 0.96 (0, 3)	NA	NA	NA	NA	NA
	3	163 - 161.2 (149, 173)	25 - 25.48 (15, 37)	5 - 6.79 (2, 13)	2 - 1.53 (0, 5)	NA	NA	NA	NA
	4	100 - 102.61 (91, 113)	28 - 26.65 (17, 37)	5 - 4.25 (1, 9)	1 - 0.46 (0, 2)	0 - 0.03 (0, 1)	NA	NA	NA
	5	25 - 23.7 (19, 28)	6 - 6.88 (2, 12)	1 - 1.24 (0, 4)	0 - 0.17 (0, 1)	0 - 0.01 (0, 0)	0 - 0 (0, 0)	NA	NA
	6	2 - 3.51 (1, 5)	3 - 1.17 (0, 3)	0 - 0.26 (0, 1)	0 - 0.05 (0, 1)	0 - 0.01 (0, 0)	0 - 0 (0, 0)	0 - 0 (0, 0)	NA

Each element of the table has the format “observed frequency – expected (posterior mean) frequency (95% Credible interval).
